# Intestinal homeostasis in the gut-lung-kidney axis: a prospective therapeutic target in immune-related chronic kidney diseases

**DOI:** 10.3389/fimmu.2023.1266792

**Published:** 2023-11-01

**Authors:** Xinyin Liu, Xiaoran Wang, Peipei Zhang, Yiwen Fang, Yanyan Liu, Yueyue Ding, Wen Zhang

**Affiliations:** ^1^ Department of Nephrology, The First Affiliated Hospital of Zhejiang Chinese Medical University (Zhejiang Provincial Hospital of Chinese Medicine), Hangzhou, China; ^2^ Department of Traditional Chinese Medicine, Jiande First People’s Hospital, Jiande, Hangzhou, China; ^3^ Department of Nephrology, The First People’s Hospital of Hangzhou Lin’an District, Hangzhou, China; ^4^ The First Clinical Medical College, Zhejiang Chinese Medical University, Hangzhou, China; ^5^ Department of Geriatric, Zhejiang Aged Care Hospital, Hangzhou, China; ^6^ Department of Geriatric, Tongde Hospital of Zhejiang Province, Hangzhou, China

**Keywords:** chronic kidney disease, gut-lung-kidney axis, intestinal homeostasis, microbiome, immunity

## Abstract

In recent years, the role of intestinal homeostasis in health has received increasing interest, significantly improving our understanding of the complex pathophysiological interactions of the gut with other organs. Microbiota dysbiosis, impaired intestinal barrier, and aberrant intestinal immunity appear to contribute to the pathogenesis of immune-related chronic kidney diseases (CKD). Meanwhile, the relationship between the pathological changes in the respiratory tract (e.g., infection, fibrosis, granuloma) and immune-related CKD cannot be ignored. The present review aimed to elucidate the new underlying mechanism of immune-related CKD. The lungs may affect kidney function through intestinal mediation. Communication is believed to exist between the gut and lung microbiota across long physiological distances. Following the inhalation of various pathogenic factors (e.g., particulate matter 2.5 mum or less in diameter, pathogen) in the air through the mouth and nose, considering the anatomical connection between the nasopharynx and lungs, gut microbiome regulates oxidative stress and inflammatory states in the lungs and kidneys. Meanwhile, the intestine participates in the differentiation of T cells and promotes the migration of various immune cells to specific organs. This better explain the occurrence and progression of CKD caused by upper respiratory tract precursor infection and suggests the relationship between the lungs and kidney complications in some autoimmune diseases (e.g., anti-neutrophil cytoplasm antibodies -associated vasculitis, systemic lupus erythematosus). CKD can also affect the progression of lung diseases (e.g., acute respiratory distress syndrome and chronic obstructive pulmonary disease). We conclude that damage to the gut barrier appears to contribute to the development of immune-related CKD through gut-lung-kidney interplay, leading us to establish the gut-lung-kidney axis hypothesis. Further, we discuss possible therapeutic interventions and targets. For example, using prebiotics, probiotics, and laxatives (e.g., Rhubarb officinale) to regulate the gut ecology to alleviate oxidative stress, as well as improve the local immune system of the intestine and immune communication with the lungs and kidneys.

## 1 Introduction

Genetic and environmental factors play a role in the pathogenesis of immune-related chronic kidney diseases (CKD), such as immunoglobulin A (IgA) nephropathy (IgAN) (1, 2), anti-neutrophil cytoplasmic antibody (ANCA)-associated vasculitis (AAV) (3, 4), and systemic lupus erythematosus (SLE) (5, 6). Gastrointestinal symptoms are rare in the early stages of these diseases, although mild diarrhea occurs in patients with IgAN (7). The discovery of the gut-kidney axis establishes the relationship between disruption of gut homeostasis and CKD onset and progression.

The gastrointestinal tract, hosting the gut microbiome, is protected by a large number of immune cells and structures for intestinal homeostasis. These structures remain in direct contact with various microbial communities in the environment (8, 9). With increased permeability of the gut epithelium, pathogens and foreign antigens enter systemic circulation, leading to CKD progression. This process is regulated by the gut microbiota and immune cells (10).

Furthermore, respiratory tract infection is the most common exogenous factor in acute exacerbations of CKD (11, 12), resulting from immune and inflammatory responses induced by pathogen-associated molecular patterns (PAMPs). The proposal of the “gut-lung” axis suggested multidirectional interactions of the lung, kidney, and gut (13). Thus, further research was required to confirm the existence of interactions between the gut, lungs, and kidneys, contributing to the pathogenesis of immune-associated CKD.

**Figure 1:**
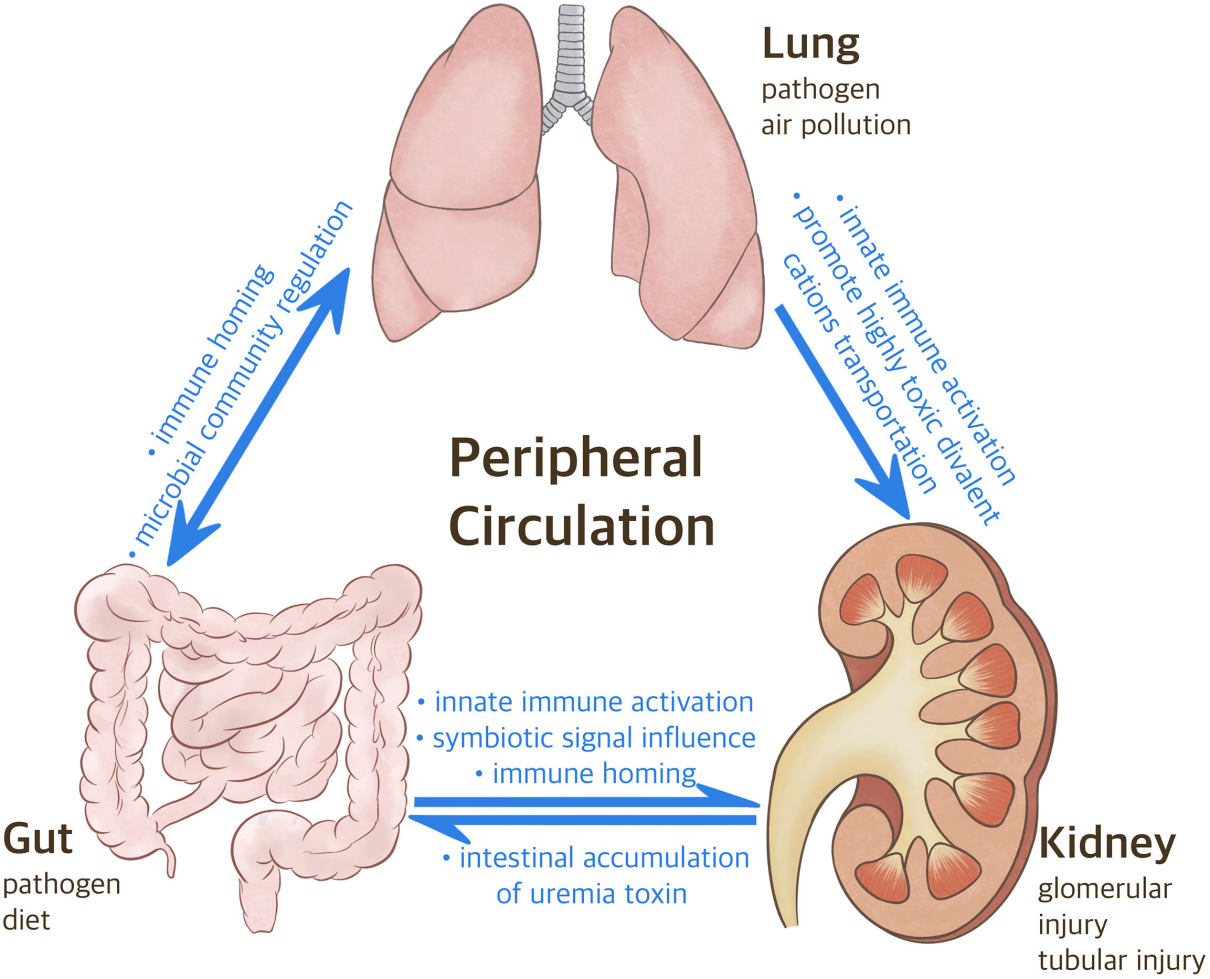
The Correlation between Gut-Lung-Kidney. Following the inhalation of various pathogenic factors (e.g., particulate matter 2.5 mum or less in diameter, pathogen) in the air through the mouth and nose, considering the anatomical connection between the nasopharynx and lungs, gut microbiome regulates oxidative stress and inflammatory states in the lungs and kidneys. Meanwhile, the intestine participates in the differentiation of T cells and promotes the migration of various immune cells to specific organs. CKD can also affect the progression of lung diseases via heavy metal.

This review aims to provide an up-to-date qualitative synthesis of recently published literature related to these organs and discusses the underlying pathophysiological mechanisms of the gut-lung-kidney axis in several diseases. Here, we focus on the connection between gut microbiota and innate and adaptive immunity, and propose possible treatment methods based on the improved understanding of the gut-lung-kidney axis. We made a graphical abstract of the gut-lung-kidney axis for clarity (**Figure 1**).

## 2 Intestinal homeostasis and human health

### 2.1 Intestinal microbiota

The human gut microbiota, coexisting in complex interdependence, is an important ecosystem comprising bacteria, fungi, archaea, viruses, and protozoa (14, 15). The intestinal flora is dominated by *Firmicutes* and *Bacteroides*, with relatively few *Proteobacteria*, *Actinobacteria*, *Fusobacteria*, and *Verrucomicrobia phyla* (16). *Bacteroidetes*, *Firmicutes*, and *Bifidobacteria* help ferment soluble dietary fiber that cannot be degraded by human digestive enzymes into short-chain fatty acids (SCFAs) (17). SCFAs are a group of fatty acids with fewer than six carbons, with acetic acid (C2), propionic acid (C3), and butyric acid (C4) accounting for more than 95% of all SCFA in the gut (18). Functions performed by SCFAs include providing approximately 10% of the daily energy required by the human body, maintaining glucose homeostasis, and reducing cholesterol levels (19–23).

SCFAs participate widely in innate immune response and can activate orphan G-protein-coupled receptors (GPRs), GPR41, and GPR43 (24). GPR43 enhances production of interleukin-18 (IL-18) to promote colonic epithelial repair through the regulation of NACHT, LRR, and PYD domain-containing protein 3 (NLRP3) inflammasome (25). In addition, SCFAs facilitate neutrophil cell migration to enhance phagocytosis (26). Another study suggested that SCFAs inhibit intestinal macrophage production of pro-inflammatory mediators (e.g., nitric oxide (NO), IL-6, and IL-12) via histone deacetylase (HDAC) inhibition (27).

In addition, SCFAs are involved in adaptive immune response and can transform immature T cells into Th1 or Th17 cells based on different cytokine environments, and increase the production of IL-17, a key cytokine in inflammation (18, 28).

In summary, the gut microbiota maintains intestinal integrity through SCFAs, mainly by modulating immune responses.

### 2.2 Intestinal immunity

#### 2.2.1 The small intestine

The small intestinal epithelium has a single layer mainly composed of columnar epithelial cells (29), expressing numerous pattern recognition receptors, including toll-like receptor 5 (TLR5) (30), TLR1, TLR2, TLR3, and TLR9 (31). Further, these cells promote the expression of chemotactic factors for myeloid cells, lymphoid cells, and neutrophil chemokines, following specific stimulation to initiate and coordinate inflammatory responses (32, 33). The intestinal epithelium contains intraepithelial lymphocytes (IELs) (34), which are almost all T cells. On stimulation, these cells express interferon-γ (IFN-γ) and keratinocyte growth factor to play protective and pathogenic roles during inflammation (35–37).

The lamina propria underlies the intestinal epithelium; within this layer, αβ T-cell receptor-positive T cells are the most commonly found lymphocytes (38). Subsets within this population have drastically different functions; CD4+ and CD25+ regulatory T cells in the lamina propria can inhibit T-cell proliferation, cytokine production, and development of colitis (39). In contrast, lamina propria CD4+ T cells secrete both IL-17 and IL-22, and are associated with intestinal inflammation (40, 41). Furthermore, lamina propria dendritic cells (LPDCs) play a significant role in determining whether a particular antigen receives an inflammatory or anti-inflammatory response. CD103^−^ LPDCs promote inflammation and increase the expression of inflammatory mediators, such as tumor necrosis factor-α and IL-6, following stimulation with TLR ligands (42). Meanwhile, CD103 LPDCs prevent excessive inflammation (43).

Innate lymphoid cells (ILCs) can be classified into three broad groups (44). Group 3 ILCs are important intestinal sources of IL-17 and IL-22 and bidirectionally regulateintestinal damage (9).

#### 2.2.2 The large intestine

B cells are the predominant lymphocytes in the lamina propria of the large intestine (38). Lamina propria B cells secrete dimeric IgA, which is transcytosed by epithelial cells into the gut lumen through the action of the polymeric immunoglobulin receptor (45, 46). Antigen-specific IgA can be generated during an intestinal infection (47). Intestinal IgA directly controls the composition of the intestinal microbiome (48). Goblet cells, another class of specialized epithelial cells, represent approximately 15% of cells found in the large intestinal epithelium (49, 50) and can produce the antimicrobial peptides Ang4, RegIIIγ, and RegIIIβ (51, 52). In addition, goblet cells may transfer antigens acquired from the intestinal lumen to dendritic cells in the lamina propria (53).

In summary, the intestinal local immune function and gut microbiota collaborate to maintain intestinal homeostasis.

#### 2.2.3 Leaky gut

Researchers have found that the disruption of intestinal barrier due to various pathological factors, leads to increased permeability of the gastrointestinal epithelium. Initially, prohibited substances (e.g. microorganisms, microbial products, and food antigens) intrude into the systemic circulation and lead to intestinal inflammation; this process is also known as “gut leaky” (54).

Gut leaky may cause a series of immune-related diseases, such as systemic lupus erythematosus (SLE) (55) and ANCA-associated vasculitis (AAV) (56).

In addition, the long-term existence of the pathological condition can also affect the remodeling of other organ tissues, which leads to fibrosis and damage to organ function (e.g. liver and kidneys) (57, 58).

## 3 The gut-kidney axis

### 3.1 The microbial pathway

The main microbiota involved in the gut-kidney axis produce SCFAs and urinary toxin solutes.

In healthy individuals, amino acid metabolites are produced following diet protein metabolism and are then converted into urinary toxin solutes. Several reviews have summarized that gut microbiota is the potential source of uremic toxins, including p-Cresyl sulfate (PCS), indoxyl sulfonate (IS), indole-3-acetic acid, trimethylamine N-oxide (TMAO), and phenylacetylglutamine (PAGIn) (59, 60). The butyrate-producing bacteria, *Roseburia*, *Faecalibacterium*, *Clostridium*, *Coprococcus*, and *Prevotella*, are reduced in end-stage renal disease (ESRD) (61). Patients with ESRD have significantly altered gut microbiota compared with individuals with normal kidney function, with a larger number of bacterial families producing urease, uratase, indole, and p-cresol-forming enzymes (62). Li et al. showed that butyrate-derived fecal bacteria in the intestines of patients with CKD were significantly decreased and were not geographically related. Butyrate reduces renal inflammation and serum levels of uremic toxins, which are partially mediated by GPR-43 signaling (62, 63).

Changes in the gut microbiota lead to poor prognoses in patients with CKD. Lack of dietary fiber or excessive protein intake can produce excessive cresol sulfate and indole sulfate (64). Decreased renal function causes the accumulation of large amounts of uremic solutes in the plasma (65, 66). Indole sulfate can cause systemic vascular inflammation, endothelial dysfunction, and vascular calcification (67). In addition, PCS induces insulin resistance in mice with normal renal function via activation of the ERK1/2 pathway (68). Indole sulfate and PCS increase expression of the angiotensin 1 (AT1) receptor and reduce expression of the AT2 receptor in semi-nephrectomized mice, thereby activating the renal renin-angiotensin-aldosterone system and significantly increasing transforming growth factor β (TGF-β) expression. This induces the transformation of renal tubular epithelial-mesenchymal tissue, which is a key factor in renal fibrosis (69), and ultimately exacerbates kidney damage (70). Plasma proteins have high binding rates to PCS and indole sulfate, making them difficult to identify via dialysis. They remain in circulation for a long time and are an important factor in the poor prognosis of patients with ESRD (71, 72). Unlike PCS and IS, TMAO and PAGIn are easily eliminated through hemodialysis; regardless, they are important uremic toxins. TMAO is produced via the products of intestinal bacterial metabolism and subsequent oxidation after dietary intake, and participates in the onset of atherosclerosis and cardiovascular disease (CVD) (73). PAGIn is the product of phenylalanine through gut microbiota metabolism, and it may increase the risk of thrombosis and CVD by stimulating platelets (74).

As renal function decreases, the colon replaces the kidneys as the main site for the excretion of urea and uric acid (75). In patients with CKD, the concentration of nitrogenous urea in the blood increases; subsequently, nitrogen moves freely from the blood to the digestive tract lumen, where it is converted into ammonia and carbon dioxide by urease. A significant positive correlation was found between blood urea concentration and total and free ammonia, thereby increasing the pH of intestinal fluid and disrupting intestinal homeostasis (76). Two studies by Waziri et al. suggested that continuous exposure of colonic epithelial cells to urea can disrupt epithelial barrier function via depletion of intestinal tight junction proteins, such as claudin 1, occludin, and zonal occludens 1 (77, 78). An increase in intestinal permeability leads to increased levels of endotoxins and bacterial products in the systemic and lymphatic circulations of patients with CKD, leading to chronic and systemic inflammation (79). This finding was confirmed in a study showing bacterial DNA in the extraintestinal region in the blood and mesenteric lymph nodes of uremic model rats, with increased levels of hsCRP and IL-6 in the plasma (80). Leaky gut causes translocation of lipopolysaccharide (LPS) and (1→3)-β-D-glucan (BG) into the circulation, activating pro-inflammatory and fibrotic pathways, leading to organ fibrosis, and exacerbating the progression of CKD (58).

In summary, the imbalance of intestinal microorganisms leads to the intestinal environment destruction and the urinary toxin solutes residual, ultimately cause the inflammation response, vascular damage and renal function damage.

### 3.2 The immune pathway

Another pathway connecting the intestines and kidneys is mediated by the immune system.

Soluble urokinase-type plasminogen activator receptor (suPAR) is an inflammatory signal with multiple biological effects expressed in various cells, including immunocompetent and endothelial cells and podocytes (81, 82). Earlier study suggested its concentration depends on the “activation” level of the immune system (81). Genua’s experiment in a mouse model of colitis found that suPAR increased irritability, prevented macrophage polarization to M1, and inhibited production of inflammatory factors in the intestinal inflammatory state (83).

In contrast, Ham et al. proposed that immature myeloid cells in the body’s bone marrow can cause a pathological increase in suPAR, and that transplantation of immature myeloid cells into healthy mice can induce proteinuria. These findings were indicative of a relationship between suPAR and CKD (84). Wei et al. believed that elevated levels of suPAR can activate the cell movement-promoting αvβ3 integrin, leading to the disappearance of foot processes and proteinuria (82).

Earlier studies suggested a specific association of high serum suPAR levels with primary focal segmental glomerulosclerosis (FSGS) (85). However, subsequent studies have confirmed that serum suPAR levels are associated with proteinuria levels and estimated glomerular filtration rate (eGFR) in primary non-FSGS glomerulonephritis, autoimmune diseases, and secondary glomerulonephritis (86), and with eGFR in patients with autosomal dominant polycystic kidney disease (87), especially those with FSGS and lupus nephritis (LN) (88). A follow-up study involving 3,683 patients suggested that higher plasma suPAR levels were independent risk factors for CKD (89). Furthermore, SuPAR is associated with glomerulonephritis and may serve as a common biomarker of glomerulonephritis (89).

In summary, the inflammatory state of the intestine may lead to renal inflammation by upregulating suPAR expression.

## 4 The gut-lung axis

The physiological distance between the intestine and lungs is relatively large, and researchers have focused on achieving long-distance communication.

### 4.1 The microbial pathway

Although the biomass of the lung microbiota is significantly lower than that of the intestinal microbiota in healthy individuals (90), the lungs and intestines have similar bacterial phyla, mainly Firmicutes and Bacteroides, followed by Proteus and Actinomyces (91). The main fungal phyla identified in the lungs are Ascomycetes and Microsporidia (92, 93).

Results of some preclinical trials suggest that changes in the pathophysiological state of the intestines or lungs can lead to changes in mutual microbiota, which closely related to inflammation. Rat fecal transplantation negatively regulates the abundance of the original intestinal microbiota and positively regulates that of the lung microbiota, thereby restoring them to a normal state (94). Conversely, acute pneumonia induced by LPS in mice affects microbiota in the blood and cecum (95).

Comparable results have been observed in both human and animal experiments. A high abundance of enterogenic *Bacteroides*, strongly associated with tumour necrosis factor alpha (TNF-α), was found in the alveolar lavage fluid of patients with acute respiratory distress syndrome (96). According to the above studies, gut microbiota had a transient response to lung damage, which may be related to the overload caused by the temporary translocation of bacteria to the intestine through circulation. However, this does not explain the improvement in pulmonary flora following fecal bacterial transplantation.

Another study on plasma metabolomics of patients with acute respiratory distress syndrome (ARDS) suggested that the increased mortality rate in ARDS was associated with high levels of plasma phenylalanine and its gut microbiota metabolite PAGIn. High levels of PAGIn are associated with thrombosis, which is one of the lethal pathological mechanisms of ARDS (74), indicating that the progress of ARDS involves gut ecology.

### 4.2 The immune pathway

Further research revealed the involvement of the local immune system in the lungs and intestines.

Local immunity in the lungs and intestines plays a vital role in organ protection. According to Samuelson’s hypothesis, the gut microbiota can be transported to gut-associated lymphoid tissue via dendritic cells (DC) and cross the intestinal barrier (97). Bacterial products can activate innate immune signaling events and trigger inflammatory reactions via the TLR pathways (97).

In addition, many T cells reside in the intestinal epithelium and lamina propria. On activation of the Peyer and mesentery lymph nodes potentiated by chemokine receptor 9 (CCR9) and integrin α (4) β (7), T cells are recruited into the intestinal mucosa (98).

Mikhak et al. suggested that DCs in the lungs activate antigen-specific CD4+ T cells and guide lung-specific homing via CCR4 to enhance immunity (99). Targeted immune responses help adjust the balance of the immune system.

Ruane et al. found cross-organ communication between the lungs and intestine. To guide the migration of T cells to the gastrointestinal (GI) tract, lung DCs activate TGF-β and retinoic acid receptor signaling pathways to upregulate the intestinal homing integrin α4β7 (100).

Furthermore, the gut microbiota is involved in phenotypic changes and migration of T cells. Intestinal segmented filamentous bacteria are symbiotic bacteria that colonize the ileum of most animals, including humans, and regulate polarization of CD4+ T cells into Th17 cells. If the lungs are in a highly inflammatory state, the level of CCL20 (a ligand of CCR6) increases, prioritizing the recruitment of Th17 cells into the lungs (101). Th17 cells aid in protection against pulmonary fungal and *Staphylococcus aureus* infections (102, 103).

In summary, the immune homing effects of the lungs and intestine partly via microbial community signal regulation contribute to mutual immune barriers.

## 5 Potential connections between the lungs and kidneys

The connection between the gut-kidney and gut-lung has been revealed gradually. Meanwhile, several studies suggest the potential physiological and pathological relationships between the lungs and kidneys.

As commonly known, CKD progression is closely associated with acute respiratory infection (11). Chronic opportunistic pathogens, such as Epstein–Barr virus (EBV), will lurk and and enter into the oropharynx, and indicate increased levels of renal involvement in patients with granulomatosis with polyangiitis (GPA) (104).

Large studies in multiple parts of the world also suggested that air pollutants (e.g., particulate matter 2.5 mum or less in diameter etc.) are significantly correlated with the decrease of eGFR and the incidence of CKD (105, 106).

Moreover, pulmonary disease, such as chronic obstructive pulmonary disease (COPD), can cause severe hypoxia and induce the expression of divalent metal transporter 1 (DMT-1) genes. DMT-1 is present in most organs, especially in the duodenum and kidneys. It transports divalent cationic cadmium 2+ (Cd 2+), which has significant osteotoxicity and nephrotoxicity. The harmful effects of Cd 2+ may worsen the microstructure of the bone and reduce renal function (107). A bidirectional Mendelian randomization study involving 321,047 Europeans suggested that renal dysfunction is a pathogenic factor for COPD (108).

Furthermore the intestinal metabolite PAGIn, also known as the urinary toxin solute, can increase the risk of thrombosis by stimulating platelets and simultaneously affect the progression of ARDS and CKD (59, 74).

The aforementioned studies strongly suggest a connection between the lungs and kidneys; however, the crosstalk mechanism of these two organs remains unclear.

## 6 The gut-lung-kidney axis and related diseases

Based on the studies cited above, we propose the hypothesis of the gut-lung-kidney axis, which suggests that the gut participates in the pathological mechanism between the lungs and kidneys, and that these organs interact with each other through microbiota and immune regulation. The possible underlying associations can be explained by observations from the following can be explained via the resulting diseases.

Studies on the topics regarding immune-related CKD are detailed in **Table 1**.

**Table 1:** List of studies conducted on the Gut-Lung-Kidney Axis.

Author	Year	Ref. Number	Related diseases	Species	Mechanism
Zhao J	2023	197	AKI-CKD	Human	There is a substantial correlation between Coprococcus2, α-hydroxybutyrate and the incidence rate of sepsis.
Sasaki K	2021	202	AKI-CKD	Mice	IRF4 expression is inhibited in sepsis, thereby macrophages polarize towards M1, the maintenance of inflammatory state leading to the occurrence of ALI.
Chen Q	2022	201	AKI-CKD	Mice	IRF4 expression is inhibited in sepsis, thereby macrophages polarize towards M1, the maintenance of inflammatory state leading to the occurrence of ALI.
Sasaki K	2022	203	AKI-CKD	Mice	IRF4-deficient mice exhibited a decrease in renal interstitial fibrosis in ischemic kidney damage compared to normal mice
Chen X	2023	200	AKI-CKD	Mice	The treg/th17 immune cell balance was disrupted in host with sepsis.
Mazza S	2020	190	COVID-19	Human	Increased fecal calprotectin levels and severe ulcerative colitis are indicative of intestinal inflammatory response related to SARS-CoV-2
Zuo T	2020	192	COVID-19	Human	The number of intestinal opportunistic pathogens (e.g. Coprobacillus, Clostridium ramosum, and Clostridium hathewayi) increased in patients with COVID-19
Abbate M	2020	193	COVID-19	Human	COVID-19 directly attack the kidneys
Pan X-w	2020	194	COVID-19	Human	High levels of ACE2 and cellular transmembrane serine proteases were successfully detected in renal tubular epithelial cells and podocytes
Su H	2020	195	COVID-19	Human	COVID-19 directly attack the kidneys
Sterlin D	2021	189	COVID-19	Human	Recirculating IgA-secreting plasmablasts with CCR10, which can efficiently home to and reside within the mucosa, were detected in infected individuals
Yeoh YK	2021	191	COVID-19	Human	Intestinal ecological imbalance can be observed in patients with COVID-19 infection, with decreased beneficial bacteria and increased opportunistic pathogens, and a long-term lack of intestinal probiotics was reported.
Abdulahad WH,	2008	136	GPA	Human	A skewed Th17 response found in ANCA-positive WG patients following stimulation with the autoantigen PR3 suggests that IL-17 is involved in disease pathogenesis
Hurtado PR	2008	153	GPA	Human	circulating anti-neutrophil autoreactive B cells are present in ANCA+ vasculitis patients, and they are capable of producing antibodies in response to CpG stimulation.
Zycinska K	2008	155	GPA	Human	The severity of Wegener’s granulomatosis disease, prevalence of gastroduodenal lesions, and the type and duration treatment seem to depend upon H. pylori infection
Lidar M	2009	104	GPA	Human	GPA patients exhibited more antibodies toward EBV viral capsid antigen IgG and EBV early antigen IgG compared to healthy people. GPA patients with increased titers of EBV viral capsid antigen IgG antibodies suffered more renal damage and had a higher Birmingham vasculitis activity score
Zycinska K	2009	144	GPA	Human	A positive PR3-ANCA test at the start of treatment, chronic nasal crusting, and Staphylococus aureus infection as risk factors for relapse
Richter AG	2009	145	GPA	Human	Pathogens were commonly grown from bronchoalveolar lavage fluid (BALF) of patients with WG and those with idiopathic pulmonary fibrosis (IPF), BALF levels of interleukin 1 receptor antagonist (IL1ra) were statistically significantly elevated. S aureus was particularly associated with patients with WG both in relapse and in remission.
Laudien M	2010	143	GPA	Human	WG patients showed significantly high rates of nasal colonisation with S. aureus. WG patients with nasal carriage of S. aureus had significantly higher endoscopically proven endonasal activity than WG patients without such carriage.
Zhao Y	2012	135	GPA	Human	There was no evidence of B-cell clones from the mucosal biopsies circulating in peripheral blood in GPA or any numerical or proportional change in B-cell subsets expressing markers of regional homing in blood in GPA.
Okamoto Yoshida Y	2010	138	GPA	Mice	IL-17A produced by TCR gammadelta T cells can induce mature granuloma formation.
Krebs CF	2016	164	GPA	Mice	Th17 cells were amplified by Citrobacter rodentium infection in the intestine, migrating from the intestinal lamina propria to the kidney via the CCL20/CCR6 axis and exacerbating renal pathology in AAV mice.
Krebs CF	2020	163	GPA	Mice	mice infected with S. aureus had persistent Th17 cells in the kidney tissue and worsened crescentic glomerulonephritis
Novak J	2002	123	IgAN	Human	Mesangial cells have a higher affinity for IgA circulating immune complexes than for uncomplexed IgA.
Smith AC	2006	117	IgAN	Human	IgA1 with high lectin binding was produced in response to mucosal HP infection in all subjects.
Eijgenraam J	2008	118	IgAN	Human	Mucosal challenge results in antigen-specific secretory high molecular weight IgA in the circulation predominantly.
Qin W	2008	122	IgAN	Human	External suppression (e.g. LPS) may causes the low Cosmc mRNA expression in IgAN.
Lai KN	2008	132	IgAN	Human	Podocytes may amplify the activation of tubular epithelial cells with enhanced TNF-alpha synthesis after inflammatory changes of human mesangial cell.
Lai KN	2009	133	IgAN	Human	Humoral factors (predominantly TNF-alpha and TGF-beta) released from mesangial cells are likely to alter the glomerular permeability in the event of proteinuria and tubulointerstitial injury in IgAN
Coppo R	2010	121	IgAN	Human	The up-regulation of TLR-4 in circulating mononuclear cells of patients with IgAN is in association with proteinuria and heavy microscopic haematuria.
Xin G	2013	129	IgAN	Human	Levels of serum BAFF were elevated in patients with IgAN and were associated with clinical and pathological features of the disease
Li W	2014	130	IgAN	Human	Overexpression of TLR9 mRNA and protein in peripheral blood mononuclear cells and elevated levels of serum BAFF may be associated with overexpression of serum IgA1
Zhang J	2020	134	IgAN	Human	Secretory IgA may induce high expression of TLR4 in human renal mesangial cells and further activate downstream signalling pathways, thereby mediating kidney damage in IgAN patients.
Baenziger JU	1980	116	IgAN	Kinetics	Only glycopeptides bearing terminal Gal or GalNAc residues can induce a conformational alteration in the receptor of liver cells which is required for uptake to occur.
McCarthy DD	2006	127	IgAN	Mice	BAFF over-expression results in increased IgA levels within the intestinal lamina propria and deposition of IgA immune complexes in the renal glomerular mesangium.
Suzuki Y	2011	119	IgAN	Mice	Disruption of mucosal tolerance result in abnormal priming and dissemination of cells to sites such as the bone marrow where they are responsible for synthesis of nephritogenic IgA.
McCarthy DD	2011	128	IgAN	Mice	The presence of commensal flora was essential for the elevated serum IgA phenotype.
Onodera T	2012	115	IgAN	Mice	In infected lungs, CXCR3 are up-regulated, and lung resident memory B cells on reinfection with distinctive phenotype will promptly differentiated into plasma cells that produced virus-neutralizing antibodies locally.
Papista C	2015	124	IgAN	Mice	Gliadin-CD89 interaction may aggravate IgAN development through induction of IgA1-sCD89 complex formation and a mucosal immune response
Ruane D	2016	120	IgAN	Mice	After intranasal immunization with inactive cholera toxin (CT), lung dendritic cells stimulated retinoic acid-dependent up-regulation of α4β7 and CCR9 gut-homing receptors on local IgA-expressing B cells.
Oh JE	2021	113	IgAN	Mice	Intranasal, but not systemic, immunization induces local IgA secretion in the bronchoalveolar space
MacLean AJ	2022	114	IgAN	Mice	The mechanism of lung resident memory B cells reactivation is mediated by Alveolar macrophages, which in part induced the expression of chemokines CXCL9 and CXCL10 from infiltrating inflammatory cells
Wang Y	2022	126	IgAN	Mice	BAFF firstly brought about the B cell responses in the local part, then subsequently in lymphoid organs. Activated B cells produced more BAFF through TLR9-IRF5 signaling pathway.
Moldoveanu Z	1990	111	IgAN	Monkey	the liver is the major site of uptake and catabolism of IgA in monkeys and possibly in humans.
Fricke W	2014	209	Kidney Allograft Rejection	Human	The decrease fo four KTRs in abundance of Anaerotruncatus, Coprobacillus etc. is related to the development of future exclusion
Wang J	2021	208	Kidney Allograft Rejection	Human	ABMR is associated with lower microbial abundance of Clostridia, Paraprevotellaceae, and Faecalibacterium, as well as higher abundance of Enterococcaceae, Coprobacillus, and Enterobacter
Peterson KS	2004	187	SLE	Human	pDCs accumulate in the glomeruli of patients with active LN
McClain MT	2005	174	SLE	Human	The latent viral protein Epstein–Barr virus nuclear antigen-1 (EBNA-1) cross-reacts with the Ro 60 kDa antibody, which is a common antibody in SLE
Henault J	2016	185	SLE	Human	DsDNA-specific IgE antibodies activated plasmacytoid dendritic cells (pDCs) in SLE, resulting in the production of substantial amounts of IFN-α (a cytokine closely related to the degree of SLE activity), and TLR9 mediated dsDNA sensing
Ogunrinde E	2019	170	SLE	Human	First-degree relatives had significantly reduced microbiome diversity compared to their controls. Bacteria in the Paenibacillus genus were reduced in first-degree relatives and SLE patients, which indicate plasma microbial translocation and microbiome may influence autoantibody development in SLE.
Azzouz D	2019	173	SLE	Human	Increase in anti-gut Ruminococcus gnavus was directly correlated with a high SLE disease activity index score
van Dam LS	2019	183	SLE	Human	SLE immune complexes (ICx) induced NET formation through Fcγ receptor signaling. SLE-induced NETs had immunogenic properties, including binding with high mobility group box chromosomal protein 1 and enrichment for oxidized mitochondrial DNA, and were involved in ICx formation.
Bai H	2021	179	SLE	Human	This positive association between LN and NO2 was also observed for south, west, and east China. In addition, we found that the short term exposure to CO and O3 was not generally associated with LN. Finally, the negative associations of LN with SO2 were found for the entire region and east China
Lartigue A	2009	172	SLE	Mice	It has been suggested that, in TLR-2 and TLR-4 deficient mice, glomerular IgG deposition and mesangial cell proliferation are significantly reduced, and antinuclear, anti-dsDNA, and anti-cardiolipin autoantibody titers are decreased.
Mu Q	2017	175	SLE	Mice	Supplementing female MRL/lpr mice with lactobacillus was found to reduce IL-6, increase IL-10, and shift the Treg/Th17 balance inside the kidney toward the Treg phenotype to protect renal function
Mu Q	2019	176	SLE	Mice	In pregnant and lactating mice, in whom indoleamine 2,3-dioxygenase enzyme is inhibited upon supplementation with lactobacilli. This reduces Treg activation and increases the levels of serum INF γ
Thim-Uam A	2020	169	SLE	Mice	Gut-leakage induced gut-translocation of organismal-molecules then enhanced the susceptibility of stress-induced apoptosis, predominantly in lupus
Issara-Amphorn J	2020	171	SLE	Mice	The pathogen molecules from leaky gut enhanced activating-FcgRs, without inhibition, through Syk, a shared downstream innate and adaptive signalling, is responsible for the hyper-responsiveness in FcgRIIb macrophages.
Yariwake VY	2021	178	SLE	Mice	Female NZBW mice exposed to concentrated ambient particles showed decreased survival, increased circulating neutrophils, early onset of proteinuria and increased kidney weight with renal cortex enlargement

### 6.1 IgA nephropathy

The pathogenesis of IgAN is currently unclear. Its onset is usually accompanied by prodromic infections of the upper respiratory tract (URT) or intestines. Most people understand the importance of IgA1 with galactose deficiency (Gd-IgA1) in circulation (109, 110).

Under physiological conditions, serum IgA1 is a common human immunoglobulin with a half-life of approximately 5 days, widely present in organs (e.g., lungs and intestine) (111).

Recently, Suzuki et al. proposed a multiple-hit hypothesis, including the production of Gd-IgA1 (Hit 1), IgG, or IgA autoantibodies that recognize Gd-IgA1 (Hit 2), their subsequent immune complex formation (Hit 3), and glomerular deposition (Hit 4) (112).

#### 6.1.1 Immune connection between prodromic infections of the URT and lungs

It is generally believed that this hypothesis is related to mucosal infections. IgAN rarely presents with obvious lower respiratory tract infection, but an emerging series of studies on mucosal vaccines has clarified the immune connection between upper respiratory tract (URT) infections and the lungs. Twenty days after primary influenza viral infection or intranasal vaccination, IgA PCs derived from resident memory B cells were observed in the submucosal areas of murine models below the bronchial epithelium (113, 114). Four days after the secondary infection, IgA PCs were detected next to the alveoli instead of the more proximal airways (114). This suggested an immune connection between URT infection and the lungs. Furthermore, IgA-producing B cells in murine models express C-X-C motif chemokine receptor 3 (CXCR3). This receptor is important for local IgA production, as CXCR3 mice have fewer IgA cells in the lungs (113–115). This suggests the possibility of URT infection participating in the course of IgAN through the lungs.

#### 6.1.2 The connection between intestinal prodromic infections and lungs

Owing to the presence of the gut-kidney axis, increased intestinal permeability in patients with IgAN may promote absorption and circulation of LPS (116). The invasion of mucosal and systemic antigens, especially proteins, glycoproteins, and viral antigens (e.g., influenza and HIV), leads to a corresponding increase in serum IgA levels with abnormal O-glycosylation (117, 118). Moreover, some studies have proposed the possibility of bone marrow-derived abnormal IgA levels (119). After the intestinal mucosa is attacked, the lungs respond accordingly. After intranasal immunization with inactive cholera toxin (CT), lung dendritic cells stimulated retinoic acid-dependent upregulation of α4β7 and CCR9 gut-homing receptors on local IgA-expressing B cells. Migration of these cells to the gut facilitated IgA-mediated protection against an oral challenge with active CT (120). This process significantly regulated by the microbiota, suggesting that lungs may participate in local immune communication through immune homing as early as the stage of intestinal infection.

#### 6.1.3 Intestinal involvement in the production of abnormal IgA1 complexes

Regarding the mechanism of abnormal IgA1 production, Coppo et al. reported that adults with IgAN have higher levels of TLR4 messenger RNA (mRNA) in peripheral hemolymph monocytes (121). Qin et al. found that LPS stimulated the activation of TLR4 in cultured peripheral B cells, inhibited cosmic mRNA expression, and resulted in galactose deficiency in IgA1 (122). When Gd-IgA1 combines with specific IgG or IgA1 antibodies to form a circulating immune complex (CIC), it remains undegraded by liver cells (123); this may be related to its excessive molecular weight and volume, which affect the normal clearance process (110). In addition, inflammation and immune activity are involved in accelerating IgA1 differentiation.

The formation of CIC also involves the intestine. A gluten diet exacerbates intestinal IgA1 secretion, inflammation, and villous atrophy, and may induce IgA1-sCD89 complex formation and mucosal immune response, ultimately potentiating IgAN development. CD89, an IgA receptor, has not been identified in human renal mesangial cells (124).

#### 6.1.4 Intestine affect the kidneys via humoral immunity

However, the mechanism underlying IgA deposition from the circulation to the kidneys remains unexplored. Some studies have suggested the presence of the CD71 receptor in human renal mesangial cells (125), which may be one of the mechanisms.

The clue to another pathway may be obtained by focusing on the B cell activating factor (BAFF). BAFF is a peripheral B-cell survival factor. Immune response against viral antigens has a positive cycle: BAFF recruits neutrophils and active B cells and generates more BAFFs through the TLR9-interferon regulatory factor 5 (IRF5) signaling pathway (126).

McCarthy et al. showed that IgA levels increased in the intestinal lamina propria, circulation, and mesangium of BAFF-overexpressing transgenic mice (127). Subsequently, a follow-up study was conducted, confirming that, in addition to BAFF levels, IgA deposition in the kidney was strongly affected by persistent symbiotic-dependent signals in the intestine (128). Some studies have shown increased serum BAFF levels in patients with IgAN, which correlated positively with disease severity, mesangial IgA deposition density, and serum BAFF and IgA1 levels (129, 130). This means that BAFF may be an important factor in gut-induced IgAN development with the involvement of the gut microbiota.

Monteiro et al. reviewed the impact of ecological changes in the gut on individuals with IgAN and found that both the abundance of gut bacteria, as well as metabolites in the blood, urine, and feces of individuals with IgAN was altered. The level of representative SCFA with anti-inflammatory effects was reduced in the feces of patients with IgAN. Disease markers of IgAN, such as proteinuria and hematuria or Gd-IgA1, were positively or negatively correlated with different bacterial abundances (131).

After circulation to the kidney, CIC can induce the proliferation of human mesangial cells, cause high expression of TLR4 in renal mesangial cells, activate the TLR4-MyD88-NF- κ B signaling pathway, release humoral factors such as TNF α, IL-6, TGF β, MCP-1, injure podocytes, change glomerular permeability, and mediate the progression of inflammation and fibrosis (132–134).

**Figure 2:**
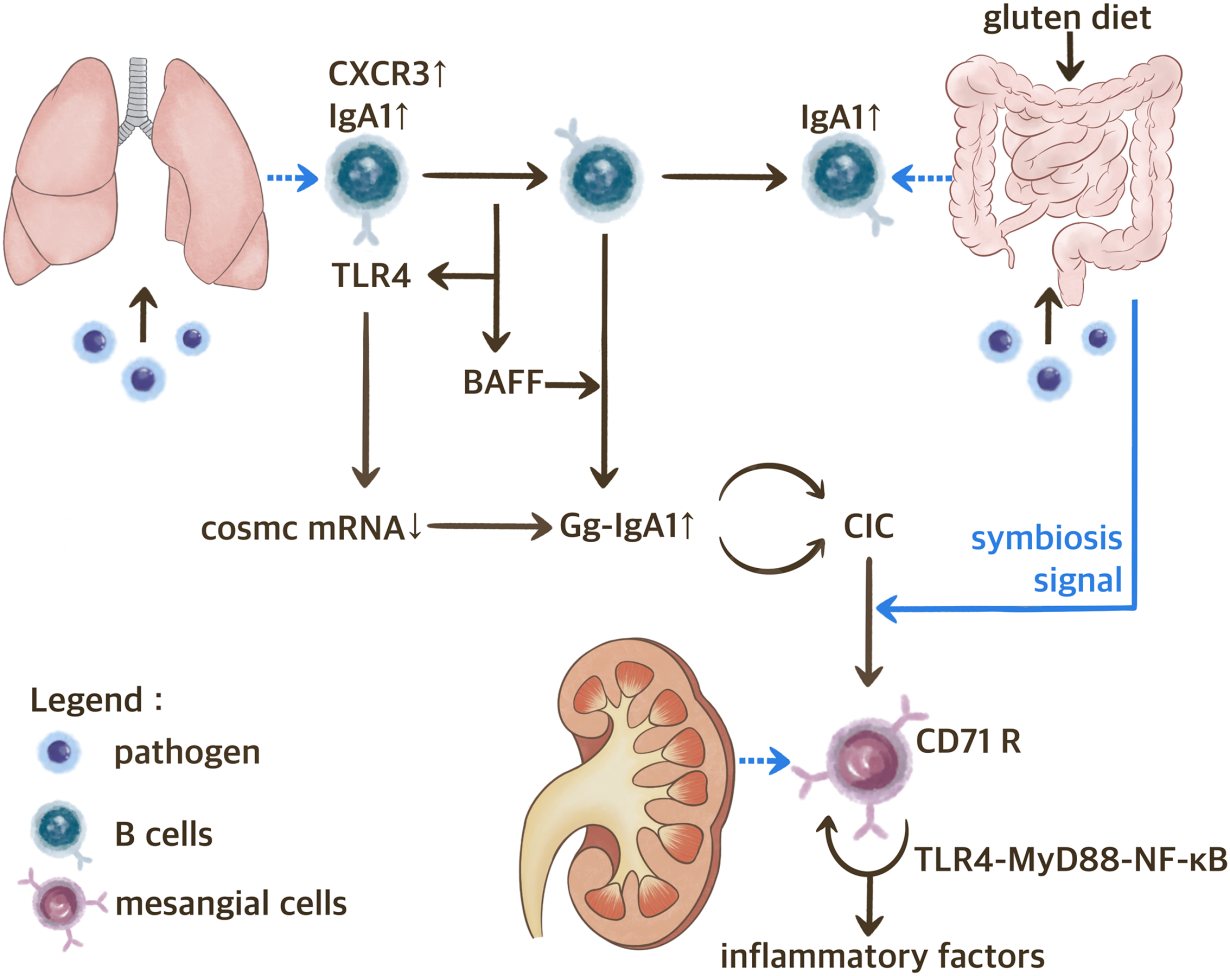
The Gut-Lung-Kidney Axis Hypothesis of IgAN. BAFF, B cell activating factor; CIC, circulating immune complex; CXCR3, C-X-C motif chemokine receptor 3; Gd-IgA1, IgA1 with galactose deficiency; TLR, toll-like receptor.

The hypothesis regarding the gut-lung-kidney axis on IgAN is shown in **Figure 2**.

### 6.2 PR3-ANCA-associated vasculitis

ANCA-associated vasculitis (AAV) is a group of chronic inflammatory systemic autoimmune diseases characterized by high circulating ANCA levels. AAV pathogenesis occurs when ANCA levels exceed the limits of human self-tolerance. This section describes the pathological mechanisms underlying Wegener’s granulomatosis (WG), also known as GPA.

The levels of Th17/Treg cells is believed to be an important link in the pathogenesis of GPA. However, the role of B lymphocytes in the pathogenesis may be limited (135). An increase in PR3-specific Th17 cells and IL-17 levels was observed in the peripheral blood of patients with GPA (136). IL-17 is an important cytokine against bacterial infection that can promote granuloma formation, indirectly attract and stimulate neutrophils, and induce ANCA production (137, 138). Tadema et al. highlighted the importance of TLR-related pathways in IL-17 production (139). Given that these pathways are part of classic innate immune defense line and an important component in identifying PAMPs, it is consistent with a previous inference that GPA is closely related to infections (140, 141).

#### 6.2.1 Respiratory infections and Th17 cells

Most studies indicate that specific pathogens are closely related to the occurrence and development of GPA. The most widely known bacteria are gram-positive bacteria (mainly *Staphylococcus aureus*), which are involved in IL-17 production in humans (142). Stegeman et al. reported a correlation between WG and *Staphylococcus aureus* mucosal infections; 95% of patients with GPA were infected with *S. aureus*, compared to only 28% of healthy people (143).

Further studies found *S. aureus* in the lungs of patients with GPA and demonstrated the benefits of antibacterial maintenance treatment. This elucidated the vital role of *S. aureus* infection in the pathology of WG (144, 145). The cell wall components of *S. aureus* include lipid teichoic acid and peptidoglycans (both TLR2 ligands), which may stimulate the release of IL-17 via TLR2-related pathways during respiratory tract infection (146–148).

In addition, gram-negative bacteria play important roles in pathogenesis. Many animal experiments have demonstrated the process of lung infection caused by gram-negative bacteria. TLR4 mediates CD4+ T cells producing IL-17 through IL-23, which is the dominant component of IL-17-inducing activity (149). The process of IL-23 participation appears to be very complex, and it involves the Jak2, PI3K/Akt, STAT3, and NF- κ B pathways (149, 150). Most studies suggest that TLR4 is upstream of IL-17; however, some studies suggest underlying interactions between IL-17 and TLR4 (151). Neutrophil extracellular traps (NETs) are released during IL-17- neutrophil recruitment, stimulating granuloma production. Histones, the main protein components of NETs, interact with TLR2 expressed on T cells, directly induce STAT3 phosphorylation, and ultimately activate Th17 cells (152). The interactions can lead to a vicious cycle of granuloma stimulation.

Excluding cell wall components, most bacteria contain CpG DNA (the TLR9 ligand), which is involved in the release of pro-inflammatory cytokines such as TNF-a and IL-1β (153). TLR4 may further interact with inflammatory cytokines to cause pulmonary fibrosis, which is a potential pathogenesis of AAV (154).

#### 6.2.2 Gut-lung balance surrounding Th17 cells

Since clinical manifestations are significantly different in patients with GPA, particularly with regards to the respiratory system and kidneys, some patients may experience gastrointestinal symptoms such as diarrhea. Therefore, it is easy to assume the immune connection between these organs.

The aforementioned studies are limited to patients with respiratory infections. Some studies have demonstrated a relationship between GI pathogenic microorganisms, especially *Helicobacter pylori* (HP), and the incidence of granulomatosis, providing a theoretical basis for intestinal involvement in the pathogenesis of GPA (155). However, data on the relationship between HP and GPA is limited. Further research is needed to elucidate the potential mechanism.

In intestinal studies, NETs have been shown to promote inflammation and apoptosis by stimulating the TLR9-mediated endoplasmic reticulum stress pathway (156) or damaging the F-actin cytoskeleton of enterocyte-like cells. This leads to intestinal epithelial damage and intestinal barrier dysfunction (157), which can cause bacterial translocation and release of intestinal inflammatory factors into the circulation (158), further affecting the composition of the gut microbiota and abundance of probiotics (159). Destruction of the gut microbiota may affect IL-17 production in the intestine (101), TLR4/NF-kB signal pathways in the lungs (160), and lead to abnormal NETs production (161), ultimately damaging the immune function of the lungs.

A study on the influenza A virus suggested its possible mechanism, proposed that the lung and intestinal mucosal state of infected mice was regulated by the balance of Th17/Treg cells. After oral administration of Houttuynia cordata polysaccharides (HCP), the balance of Th17/Treg cells was first restored in the gut mucosal-associated lymphoid tissue (GALT), followed by the lungs. HCP regulates the expression of chemokine CCL20 and CCR6 (the CCL20 receptor), promoting the specific migration of Th17/Treg cells from the gut to the lungs (162).

#### 6.2.3 Intestinal microbiota participates in gut-kidney axis migration of Th17 cells

The renal complications of GPA also involve the intestine. Krebs et al. found that mice infected with *S. aureus* had persistent Th17 cells in the kidney tissue and worsened crescentic glomerulonephritis (163). Th17 cells were amplified by *Citrobacter rodentium* infection in the intestine, migrating from the intestinal lamina propria to the kidney via the CCL20/CCR6 axis and exacerbating renal pathology in AAV mice. The cultivation of these mice under sterile conditions or antibiotic treatment significantly downregulates Th17 cells and alleviates kidney inflammation. This indicates a relationship between the gut microbiota, intestinal local immunity, and kidney involvement (164).

#### 6.2.4 EBV infection and renal complications of GPA

EBV is a common human herpes virus. After the initial infection, most patients have no symptoms; however, EBV infects the B cells through oral epithelial cells and establishes a lifelong latent infection (165).

Patients with GPA exhibited more antibodies against EBV viral capsid antigen IgG and EBV early antigen IgG compared to healthy individuals. Patients with GPA presenting with increased titers of EBV viral capsid antigen IgG antibodies suffer from increased renal damage and have a higher Birmingham vasculitis activity score (104). Due to the interconnected anatomical structure of the URT and lungs, this may indicate an underlying connection between the renal system and lungs.

### 6.3 Systemic lupus erythematosus

SLE is an autoimmune disease characterized by abnormalities in adaptive immunity (166, 167) and accompanied by destruction of innate immunity (168).

#### 6.3.1 Intestinal mucosal infection in SLE

Few studies exist on lupus-associated lung and GI injuries, especially on GI symptoms that are not clinically evident. However, in various human and animal experiments, serum PAMPs (e.g., LPS and BG) increased during the active period of lupus and were associated with leaky gut (169–171).

The close association of foreign pathogen infections with TLR-related pathways is detailed in the manuscript section on AAV. It has been suggested that, in TLR-2 and TLR-4 deficient mice, glomerular IgG deposition and mesangial cell proliferation are significantly reduced, and antinuclear, anti-dsDNA, and anti-cardiolipin autoantibody titers are decreased. This indicates the importance of pathogen infection in lupus (172).

In addition to damaging the intestinal barrier, some pathogens can expedite SLE onset through molecular simulations. Increase in anti-gut *Ruminococcus gnavus* (RG) was directly correlated with a high SLE disease activity index score (173). LPS cross-reacts with lupus anti-dsDNA antibodies (SLE-specific antibodies) (173). In addition, the latent viral protein Epstein–Barr virus nuclear antigen-1 (EBNA-1) cross-reacts with the Ro 60 kDa antibody, which is a common antibody in SLE (174).

#### 6.3.2 Intestinal ecology regulates renal immunity

Furthermore, Mu et al. confirmed that activation of LN is regulated through intestinal ecology, not only through gut leakage. Supplementing female MRL/lpr mice with lactobacillus was found to reduce IL-6, increase IL-10, and shift the Treg/Th17 balance inside the kidney toward the Treg phenotype to protect renal function (175). However, contrasting results were observed in pregnant and lactating mice, in whom indoleamine 2,3-dioxygenase enzyme is inhibited upon supplementation with lactobacilli. This reduces Treg activation and increases the levels of serum INF γ (176), an important pro-inflammatory cytokine that promotes the development of lupus (177).

#### 6.3.3 Air quality and LN

As shown by some studies, particulate matter 2.5 mum or less in diameter, which is related to the incidence and severity of lupus, leads to an increase in circulating neutrophils, proteinuria, and kidney weight; in addition, improving air quality can effectively alleviate the condition (178, 179). These findings demonstrate the relationship between the respiratory system and LN.

Some researchers believe that environmental molecules (e.g., LPS and BG) can serve as TLR-4 ligands and activate the Fc gamma receptor (FcγR) together, possibly through FcγR-TLR-4 crosstalk, thereby inducing synergistic pro-inflammatory responses (180, 181). The interaction of TLR4 and inflammatory cytokines may lead to pulmonary fibrosis in AAV and lupus patients (154). In addition, blocking spleen tyrosine kinases (the shared downstream signaling molecules of Fc gamma receptor (FcγR) and TLR-4) can alleviate lupus-induced inflammation (171).

#### 6.3.4 Intestinal ecological imbalance and air pollution co-induce LN

In SLE, autoantibodies bind to cell-expressed antigens to form an immune complex (IC). These ICs pass through FcγR and bind to inflammatory cells, causing chronic inflammation and target cell destruction. This induces the formation of NET, which is degraded and impaired due to IFN (182, 183). Abnormal NETs are difficult to clear; therefore, the body must be exposed to dsDNA for a long time to produce antibodies (183). Yun et al. demonstrated that anti-dsDNA antibodies bind directly to mesangial and proximal renal tubular epithelial cells, triggering subsequent inflammatory responses through protein kinase C and mitogen-activated protein kinases pathways, eventually inducing fibrosis (184).

Meanwhile, Henault et al. reported that dsDNA-specific IgE antibodies activated plasmacytoid dendritic cells (pDCs) in SLE, resulting in the production of substantial amounts of IFN-α (a cytokine closely related to the degree of SLE activity), and TLR9 mediated dsDNA sensing (185, 186). pDCs accumulate in the glomeruli of patients with active LN (187).

Overall, the intestinal leakage of lupus enhances the level of serum pathogen molecules (e.g., LPS and BG) and cytokines (IFN- γ and IL-6), leading to extracellular DNA exposure and production of autoantibodies and cross-reactive antibodies. Environmental molecules (e.g., PM2.5) may participate in this process through the lungs. However, the TLR-4-related pathway actively promotes inflammation and induces subsequent pulmonary and renal fibrosis. On the other hand, dsDNA-specific IgE antibodies activate pDCs of the peripheral circulation and glomerulus, increase secretion of IFN- α, and enhance renal inflammation.

### 6.4 Coronavirus disease 2019

The oral cavity and conjunctiva are common infective routes of the coronavirus disease 2019 (COVID-19). Owing to the universality of angiotensin-converting enzyme 2 (ACE2) receptors in the human body, the virus can enter the body via these routes. Following this, the immune system responds quickly, activating immune cells and molecules (e.g., secretory IgA), which spread throughout the mucosa (188). High severe acute respiratory syndrome coronavirus 2 (SARS-CoV-2)-specific serum IgA levels were detected in the mucosal secretions; meanwhile, recirculating IgA-secreting plasmablasts with CCR10, which can efficiently home to and reside within the mucosa, were detected in infected individuals (189). Increased fecal calprotectin levels and severe ulcerative colitis in some patients are indicative of intestinal inflammatory response related to SARS-CoV-2 (190). Intestinal ecological imbalance can be observed in patients with COVID-19 infection, with decreased beneficial bacteria and increased opportunistic pathogens. Yeoh et al. reported a long-term lack of intestinal probiotics (e.g. *Faecalibacterium prausnitzii*, *Eubacterium rectale*, and *bifidobacteria*) following COVID-19 infection (191). In addition, Zuo et al. found an increase in the number of intestinal opportunistic pathogens (e.g. *Coprobacillus*, *Clostridium ramosum*, and *Clostridium hathewayi*) in patients with COVID-19 (192).

Similar to the above-mentioned diseases, gut leakage may exist in COVID-19 infection, causing the virus to directly attack the kidneys (193). High levels of ACE2 and cellular transmembrane serine proteases were successfully detected in renal tubular epithelial cells and podocytes, revealing the underlying mechanism of SARS-CoV-2-related renal injury (194). The autopsy results of 26 patients who died when infected with COVID-19 provided strong evidence for this hypothesis (195). Moreover, as an important factor in the renin-angiotensin-aldosterone system, abnormal activation of ACE2 may further accelerate pro-inflammatory and fibrotic processes in the kidneys (196).

### 6.5 Acute kidney injury-CKD in sepsis

Common complications of sepsis include acute lung injury (ALI) and acute kidney injury (AKI) among others. In some patients, AKI cannot be completely reversed and it develops into CKD, which involves complex immune responses.

#### 6.5.1 Damage of intestinal barrier before the onset of sepsis

Research has found that the potential disruption of the intestinal barrier occurs prior to the onset of sepsis. A genome study on intestinal microorganisms and metabolites in patients with severe sepsis suggests that there is a substantial negative correlation between Coprococcus2 and the incidence rate of sepsis. Coprococcus2 is known as one of the butyrate-product bacteria. There is a significant correlation between α-hydroxybutyrate, a nascent biomarker for hypoxia and/or mitochondrial dysfunction, the incidence of sepsis, and the 28-day mortality rate (197).

The imbalance of intestinal homeostasis further exacerbates sepsis through gut leaky. The subsequent development of sepsis is closely related to IRF4.

#### 6.5.2 IRF4 affects the lungs and kidneys by regulating macrophage polarization

IRF4 is a transcription factor in the IRF family which is expressed in various immune cells and can regulate immune response, cell growth, and metabolism. IRF4 is a multi-effect factor. Moreover, it can promote macrophage polarization toward the M2 phenotype and macrophage apoptosis, reduce macrophage migration and inflammatory factor expression, induce fibrosis, as well as negatively regulate TLR-related pathways. Furthermore, it promotes the maturation of Th17 cells and maintains an inflammatory state of the tissue by T cell-derived IL-17 (198).

An experiment on LPS-induced sepsis mice model suggests TLR-related pathways, as principle innate immune defense lines, are extensively activated in sepsis (199). he balance of Treg/Th17 immune cells is disrupted, and a large amount of IL-17 is secreted (200).

IRF4 expression is inhibited and thereby macrophages polarize towards M1. The maintenance of an inflammatory state leads to the occurrence of ALI. After the application of Dihydroquercetin or Dexmedetomidine, IRF4 is upregulated in sepsis mice models, and macrophages polarized towards M2, producing effective anti-inflammatory effects (201, 202).

The M2 state of macrophages also promotes fibrosis of renal tissue, which is one of the core mechanisms of AKI-CKD (198).

Sasaki et al. demonstrated that IRF4-deficient mice exhibited a decrease in renal interstitial fibrosis in ischemic kidney damage compared to normal mice, confirming the core role of IRF4 in this process (203).

#### 6.5.3 Expression of intestinal IRF4 in sepsis: a blueprint yet to be elucidated

Interestingly, it has been observed in the mice models of inflammatory bowel disease, that the expression of IRF4 in CD3+T cells in the intestinal lamina propria is increased. Internal and exogenous IRF4 synergistically promotes the maturation of Th17 cells and maintains an inflammatory state in the intestine (204, 205).

This appears to be in contrast to the decrease in IRF4 levels measured in sepsis mice models. Hence, it is probable that depending on different organs/tissues, diseases, and compensatory states, the main regulatory direction of IRF4 may also be different.

Since the intestinal barrier disrupts before sepsis as mentioned above, we believe that changes in intestinal IRF4 expression and its impact on the internal environment may be important factors that have not yet been elucidated in our understanding of the mechanism of sepsis. Further experiments are needed in this regard.

### 6.6 Kidney allograft rejection

Kidney transplantation is an ideal treatment method for ESRD patients, but rejection reactions are common. Kidney allograft rejection can be classified into T-cell-mediated rejection and antibody-mediated rejection (ABMR) (206).

#### 6.6.1 Influence of gut microbiota on the outcome of kidney transplantation

Immune tolerance is crucial for ensuring graft function and survival of transplant recipients. Peripheral tolerance of T lymphocytes is the main mechanism of organ transplantation tolerance. In this process, Tregs inhibit alloreactive T lymphocytes (207).

Researchers have found an immunological association between the gut microbiome and prognosis of kidney transplantation.

According to Wang et al., there is a significant difference in the gut microbiota between kidney transplant recipients (KTRs) with ABMR and KTRs without graft rejection. ABMR is associated with a lower microbial abundance of *Clostridia*, *Paraprevotellaceae*, and *Faecalibacterium*, as well as a higher abundance of *Enterococcaceae*, *Coprobacillus*, and *Enterobacter* (208).

Another study analyzed the rectal microbiota of four KTRs who experienced rejection and compared their observations with KTRs who did not experience rejection before and after transplantation. The decrease in the abundance of *Anaerotruncatus*, *Coprobacillus* and other bacteria was related to the development of future exclusion events (209).

Clostridia can regulate Tregs differentiation (207) and is related to a lower IL-6 level (210). In addition, lower IL-17 production is associated with a higher abundance of *Faecalibacterium*, while a higher abundance of *Escherichia*, *Anaerotruncus*, *Coprobacillus*, and *Clostridium* is linked to positive regulation in IL-17 production (210).

The gut microbial community affects cytokine levels and differentiation of immune cells, thereby affecting the prognosis of transplant hosts.

#### 6.6.2 Oral microbiome and side effects of kidney transplantation

Rejection reactions could be acute or chronic. However, since they are not autoimmune diseases, kidney allograft rejection seems to have little connection with the entire respiratory tract when compared to other immune-related CKD.

According to reports, the prevalence of opportunistic pathogens, such as Enterobacteriaceae, *Pseudomonas fluorescens*, *Actinetobacter* spp., and *Vibrio* spp., is increased in the oral microbiome of transplant patients. Oral cancer is often observed in KTRs (211). Whether the higher incidence rate of oral cancer is related to the change in microbial activity in the lungs and intestines or is a result of the use of immunosuppressants after transplantation remains unclear.

## 7 New targets for treatment

At present, the mainstream treatment for pathogenic chain reactions caused by intestinal homeostasis imbalance is to restore abnormal gut microbiota to normal. Within the scope of this review, prebiotics and probiotics can be used as targeted interventions for the treatment of CKD. They can regulate intestinal microbiota, improve lipid profiles, reduce uremic toxins, maintain intestinal homeostasis, as well as reduce systemic inflammation and oxidative stress (17, 212). A survey involving 13271 Korean adults suggests that the prevalence of CKD is significantly lower in patients who take probiotic supplements than in those who do not (213).

Laxatives may be a new targeted intervention for gut microbiota in CKD. Sumida et al. demonstrated that patients with constipation have a significantly higher risk of incidental CKD and ESRD (214); the authors published a review on constipation and CKD in 2020, comparing the improving effects of various constipation drugs on CKD progression (215).

In China, *Rhubarb officinale*, a traditional Chinese herbal medicine with a laxative effect, is commonly used to treat CKD, and has a synergistic effect on angiotensin-converting enzyme inhibitors or angiotensin receptor blockers (216).

Another study conducted on 5/6 nephrectomy model rats showed that rhubarb enemas can regulate the abundance, and composition of the intestinal flora, increase SCFA levels, improve intestinal barrier damage, reduce inflammation levels, improve kidney pathology, reduce blood creatinine levels. However, the detailed mechanisms remain unclear. Further studies on the involvement of rhubarb in the gut-kidney axis are warranted (217).

The above two therapies mainly focus on the microbiota, considering the impact of local intestinal immunity on IgAN, several studies have been conducted on gut-targeted-release formulation of budesonide. Compared to systemic glucocorticoid therapy, its efficacy and safety have been effectively improved (218). The effectiveness of this drug on other immune-related CKD needs further confirmation.

## 8 Conclusions

In the field of immune-related CKD, a new concept of the gut-lung-kidney axis concept needs to be developed, which may be applied to clinical practice. Changes in intestinal homeostasis affect innate and adaptive immunities, as well as the gut-lung and gut-kidney axes, resulting in mutual interference between the intestine, lungs, and kidneys. In addition, existing data show that environmental molecules dominated by PAMPs respond to intestinal symbiotic communities through the lungs, which affect the progression of immune-related CKD.

## Author contributions

XL: Writing – original draft. XW: Writing – original draft. PZ: Writing – review & editing. YF: Writing – review & editing. YD: Writing – review & editing. YL: Writing – review & editing. WZ: Writing – review & editing.

## Glossary

**Table:**

AAV	ANCA-associated vasculitis
ABMR	antibody-mediated rejection
ACE2	angiotensin-converting enzyme 2
AKI	acute kidney injury
ALI	acute lung injury
ANCA	anti-neutrophil cytoplasmic antibody
ARDS	acute respiratory distress syndrome
BAFF	B-cell activating factor
BG	(1→3)-β-D-glucan
Cd 2+	cadmium 2+
CIC	circulating immune complex
CKD	chronic kidney diseases
COVID-19	coronavirus disease 2019
COPD	chronic obstructive pulmonary disease
CVD	cardiovascular disease
CXCR3	C-X-C motif chemokine receptor 3
DMT-1	divalent metal transporter 1
EBV	Epstein–Barr virus
eGFR	estimated glomerular filtration rate
ESRD	end-stage renal disease
FcγR	Fc gamma receptor
FSGS	focal segmental glomerulosclerosis
Gd-IgA1	IgA1 with galactose deficiency
GI	gastrointestinal
GPA	granulomatosis with Polyangiitis
GPRs	G protein-coupled receptors
HP	*Helicobacter pylori*
IC	immune complex
IELs	intraepithelial lymphocytes
IFN-γ	interferon-γ
IgAN	IgA nephropathy
IL	interleukin
IRF	interferon regulatory factor
KTRs	kidney transplant recipients
LN	lupus nephritis
LPDCs	lamina propria dendritic cells
LPS	lipopolysaccharide
IS	indoxyl sulfonate
mRNA	messenger RNA
PAGIn	phenylacetylglutamine
PAMPs	pathogen-associated molecular patterns
PCS	p-Cresyl sulfate
NETs	Neutrophil extracellular traps
SARS-CoV-2	severe acute respiratory syndrome coronavirus 2
SCFA	short-chain fatty acids
SLE	systemic lupus erythematosus
suPAR	soluble urokinase-type plasminogen activator receptor
TGF-β	transforming growth factor β
TLR	toll-like receptor
TNF-α	tumor necrosis factor alpha
TMAO	trimethylamine N-oxide
URT	upper respiratory tract
WG	Wegener’s granulomatosis
